# Mercury: Cleaner Air on the Fly?

**DOI:** 10.1289/ehp.114-a277a

**Published:** 2006-05

**Authors:** Lance Frazer

The coal industry represents more than half of America’s energy production, and DOE estimates place the recoverable reserve at more than 250 billion short tons. Coal is notorious for its drawbacks, however, including emissions of sulfur (which in the form of sulfur dioxide can react with atmospheric water to form sulfuric acid) and mercury (a known neurotoxicant). Now scientists from the Energy Research Center at Lehigh University, led by Carlos Romero, have shown that it may be possible to reduce mercury emissions by up to 70% without a lot of costly modifications, simply by optimizing boiler operation.

The USGS report *Mercury in U.S. Coal: Abundance, Distribution, and Modes of Occurrence* states, “The mercury emitted directly from power plants is not considered harmful; however, in the natural environment, mercury can go through a series of chemical transformations that convert elemental mercury to a highly toxic form [methylmercury] that is concentrated in fish and birds.” In large doses, methylmercury can cause mental retardation, seizures, cerebral palsy, and death in humans. Though some mercury is removed by cleaning the coal before burning, and more is recaptured in the stack, the EPA estimates that coal-fired power plants release 40 to 52 tons of mercury each year.

Currently, according to Romero, the industry relies on techniques such as injecting activated carbon into the flue gas stream to adsorb the mercury. One costly problem with this approach is that a typical 250-megawatt power plant can use significant amounts of activated carbon, at a cost of about 50¢ per pound.

The goal of Romero’s optimization technique is to leave more unburned carbon in the fly ash, the residue left after combustion of pulverized coal. The more carbon the fly ash contains, the better able it is to capture oxidized mercury (formed when mercury combines with chlorine, also found in coal). It’s not clearly understood why fly ash captures mercury, Romero admits, and more research is being done to explain this interaction.

“Our testing has shown that if you lower the amount of excess air in the boiler [and thus lower the flue gas temperature], you increase the level of unburned carbon,” he explains. “You can also increase the level of unburned carbon by grinding the coal more coarsely.” Results vary depending on the type of coal used and the boiler configuration.

Further tweaking will address a couple of potential drawbacks to the approach. Fly ash is used in Canada and the United States in the manufacture of cement, but due to the physical qualities of the unburned carbon, fly ash can contain only a certain amount (about 4–6%). Plus, flue gas temperatures must not be lowered too dramatically, says Romero, lest acids form in the gas, creating corrosion in the smokestack.

Under the Clean Air Interstate Rule of March 2005, the EPA has mandated a 23% reduction of mercury by 2010 and a 69% reduction by 2018. Romero thinks some boilers could achieve the first reduction through boiler optimization. “The sixty-nine percent [reduction] will be tough to achieve with combustion optimization,” he says, “but I believe this approach can be a valuable tool in industry’s efforts to reduce mercury emissions.”

George Offen, senior technical leader for air emissions and combustion product management at the Electric Power Research Institute, says that while this may be a low-cost approach to achieving moderate reductions in mercury emissions, larger plants will retrofit with other technologies to meet the requirements of the Clean Air Interstate Rule. “However,” he adds, “many smaller plants, or those located far away from locations that use fly ash in concrete, could find this process very attractive.”

## Figures and Tables

**Figure:**
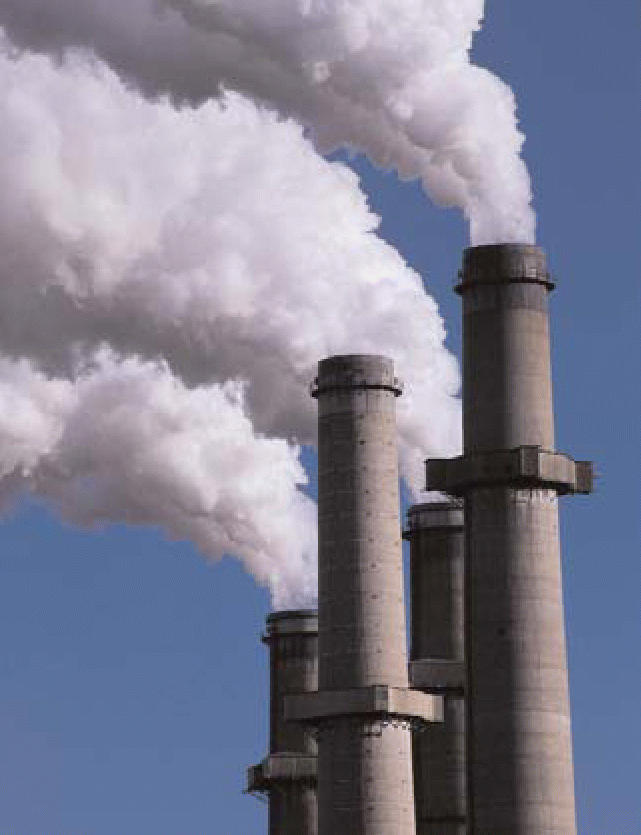
Cutting coal’s costs. New boiler configurations may lead to fewer mercury emissions.

